# Severe Legionnaires disease complicated by multi-organ dysfunction in a previously healthy patient: a case report

**DOI:** 10.1186/1757-1626-2-9151

**Published:** 2009-12-07

**Authors:** Kays Kassha, Issam Abuanza, Samer A Hadi, Roy Hilton

**Affiliations:** 1 Surgical Department, Glan Clwyd Hospital, Rhuddlan Road, Rhyl, LL18 5UJ, UK; 2 A&E Department, Glan Clwyd Hospital, Rhuddlan Road, Rhyl, LL18 5UJ, UK; 3 Medical Department (COTE) Glan C lwyd Hospital, Rhuddlan Road, Rhyl, LL18 5UJ, UK

## Abstract

A case of a previously healthy lady with community-acquired pneumonia who progressed to severe acute respiratory distress syndrome, acute renal failure, disseminated intravascular coagulation and sepsis is reported. Treatment with antibiotics and various modes of mechanical ventilation in the intensive care unit were successful. A urinary legionella antigen test was positive for *Legionella pneumophila*.

## Introduction

*Legionella *is a cause of both community and nosocomial pneumonia. A recent study found that it accounted for 2 to 9% of community-acquired pneumonia. Unless anti-*Legionella *antibiotics are selected, the mortality of legionellosis has been reported to be 60-70%; the use of appropriate antibiotics decreases mortality to 10-20%. We report a case of severe *Legionella *pneumonia that was successfully treated with antibiotics, corticosteroid and various modes of mechanical ventilation in the intensive care unit.

## Case presentation

A 58-year-old white Caucasian British lady, came to the emergency department with a 2 day history of high fever (38.6°C) shortness of breath, productive cough and diarrhoea 3 days after she had arrived from New Jersey.

In the emergency department, her respiratory rate was 21/min, blood pressure 88/57 mmHg, and heart rate 116/min and regular and Arterial oxygen saturation was 77% on room air and increased to 90% on 100% oxygen via a face mask. Rales were present in one third of the lung fields bilaterally and Percussion revealed dullness at the base of the right lung, with poor air entry in the right lower base. Additionally, her blood gas results were her blood gas results were, pH: 7.49, PaO2: 4.9 mmHg, PaCO2: 3.8 mmHg, HCO-3: 24 mM, BE: -0.5 mM and SaO2 77%. Her CURB score surprisingly was 1, one point for Low blood pressure.

**Figure 1:**
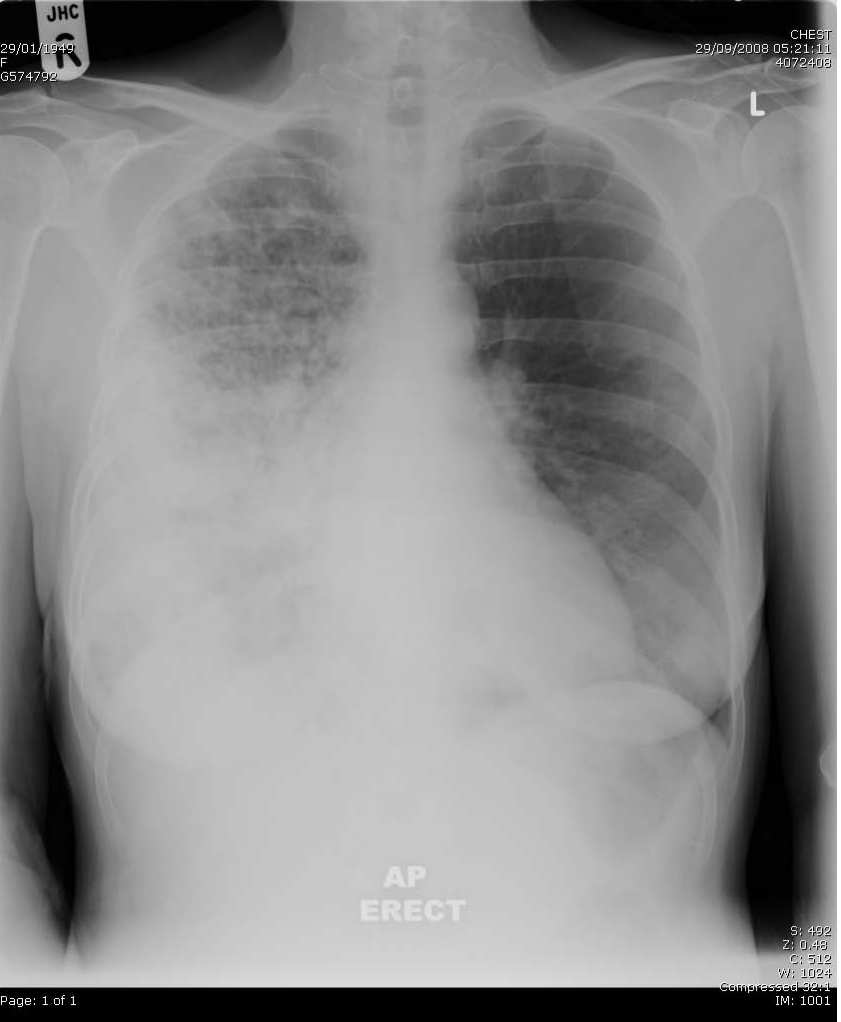
**Chest X-ray on admission which showed right lower lobe consolidation'**.

A chest radiograph showed extensive consolidation present throughout the right lung (Figure 1) and there was also a little patchy consolidation in the left mid and lower zones.

Due to septic shock, acute respiratory distress syndrome and disseminated intravascular coagulation The patient was admitted to the ITU and she was placed on CPAP initially and inotropes were started. The patient's hypoxemia increased, and there was marked progression of the bilateral shadows on chest X-ray, suggestive of ARDS. Hence, the patient was placed on mechanical ventilation, steroid infusion and an antibiotic regime of clarithromycin, ceftriaxone, rifampicin and clindamycin commenced. Because she did not show any signs of improvement, she was transferred to another hospital for ECMO (Extracorporeal membrane oxygenation), which she had for 5 days. On hospital day 6, her urine output was low and so she was put on RRT (Renal replacement therapy).

On the seventh day after admission, *Legionella *antigen was detected in the urine, the diagnosis of *Legionella *pneumonia was confirmed. Also, Bacteriological examination of sputum disclosed *Legionella pneumophila*. All her antibiotics were stopped except ceftriaxone, and she was continued on prednisolone for 3 more weeks.

After the 21^st ^hospital day, the radiographic findings gradually improved, the patient was taken off the ventilator and placed on CPAP. Moreover, her kidney functions recovered and RRT was stopped and she was moved to a respiratory ward, where she remained for another 4 weeks during which she had nutritional and physiotherapy support. The lady was discharged with almost full range of mobility and independency in day 70^th^. Table 1 shows the clinical course of the present case.

**Table 1:** Biochemistry

Blood Tests	Day1	Day2	Day10	Day15	Day20	Day30	Day45	Day60	Day70	Day75
**Wcc**	1.6	20.3	11.4	5	4.8	8.5	11.2	9.1	14.8	10.5
**Urea**	8.5	7.4	15.1	21.4	17.6	8.9	3.9	2.1	2.6	2

## Discussion

Legionnaire's disease is due to *L pneumophila*, which causes an atypical pneumonia. The natural habitat for *L pneumophila *is fresh water or protozoan biofilms, in which the bacteria parasitize and proliferate inside the protozoa. Once the water is transferred into man-made water reservoirs, the *Legionella *organisms proliferate. *Legionella *is transmitted to humans via inhalation of colonized aerosols or droplets, which are produced by air conditioners, cooling towers and condensers, water fountains, shower heads, faucets, whirlpools, ice machines, spas, nebulizers, and humidifiers. Advanced age, immunocompromised state, cigarette smoking, chronic lung disease, and male sex are all risk factors for Legionnaire's disease [1].

### Clinical manifestations

The signs and symptoms are nonspecific and are similar to those of an atypical pneumonia, but more severe. The incubation period is 2 to 10 days; during this time, the signs and symptoms are typically mild. The cough at first is mild and only slightly productive. symptoms are often prominent with diarrhea, nausea, vomiting, and abdominal pain. Examination of the lungs may initially reveal focal rales that will eventually become diffuse. Findings on an initial radiograph of the chest may be normal, but eventually a pulmonary infiltrate will develop [2,3].

• Cough - 41 to 92 percent

• Chills - 42 to 77 percent

• Fever > 38.8°C - 88 to 90 percent

• > 40°C - 20 to 62 percent

• Dyspnea - 25 to 62 percent

• Headache - 40 to 48 percent

• Myalgia/arthralgia - 20 to 40 percent

• Diarrhea - 21 to 50 percent

• Nausea/vomiting - 8 to 49 percent

• Neurologic abnormalities - 4 to 53 percent

• Chest pain - 13 to 35 percent

### Diagnosis

Four tests are predominantly used: culture of specimens, direct fluorescent antibody test, urine antigen test, and serum antibody assay. The urine antigen test is highly specific, provides rapid results, and is particularly useful because the fact that test positivity can persist for days even during administration of antibiotic. The major disadvantage of the urinary antigen test is that it is specific for L. pneumophila, serogroup 1 only. However, the vast majority of Legionnaires' disease cases from the community are caused by this species and serogroup. specifity of suptum culture is high, however, obtaining an adequate sputum specimen can be difficult. The test for serum antibodies to *Legionella *has a high specificity, but the lowest sensitivity, in part because a 4-fold increase in antibody levels is necessary for detection of the antibodies. Additionally, an antibody response may not be detectable until after 4 to 12 weeks of infection. DNA amplification by polymerase chain reaction (PCR) of Legionella has been reported from patients with pneumonia using throat swab specimens, bronchoalveolar lavage (BAL), urine, and serum. To date, clinical experience has not shown PCR to be more sensitive than culture, and therefore the Centers for Disease Control and Prevention (CDC) does not recommend the routine use of genetic probes or PCR for the detection of Legionella in clinical samples [4-6].

### Treatment

The treatment of community-acquired pneumonia without the benefit of diagnostic testing should include empiric treatment for infections caused by several microorganisms, including *Legionella *species. The American Thoracic Society and Infectious Diseases Society of America consensus committees on community-acquired pneumonia recommend empiric therapy with azithromycin, either as monotherapy or combined with a β-lactam agent. Monotherapy with a respiratory quinolone such as levofloxacin also is acceptable [7,8].

### Patient's perspective

''Started with sever Flu-like symptoms in mid-September, remember nothing until mid- October. Loss of use of limbs continues with tracheotomy made situation very frightening. However, mostly recovered apart from weakness of limbs generally. On- going stomach problems still to be investigated by the GP. My thanks to staff a Glan Clwyd Hospital and at Leicester (ECHO machine) who probably saved my life".

## Consent

Written informed consent was obtained from the patient for publication of this case report and accompanying images. A copy of the written consent is available for review by the Editor-in-Chief of this journal.

## Competing interests

The authors declare that they have no competing interests.

## Authors' contributions

IA: analyzed and interpreted the patient data, performed the physical examination, and was a major contributor in writing the manuscript. KK: major contributor in writing the manuscript. SAH: major contributor in writing the manuscript. RH: major contributor in reading and correcting any misspellings in the manuscript. All authors read and approved the final manuscript
